# Predicting 1-Hour Thrombolysis Effect of r-tPA in Patients With Acute Ischemic Stroke Using Machine Learning Algorithm

**DOI:** 10.3389/fphar.2021.759782

**Published:** 2022-01-03

**Authors:** Bin Zhu, Jianlei Zhao, Mingnan Cao, Wanliang Du, Liuqing Yang, Mingliang Su, Yue Tian, Mingfen Wu, Tingxi Wu, Manxia Wang, Xingquan Zhao, Zhigang Zhao

**Affiliations:** ^1^ Department of Pharmacy, Beijing Tiantan Hospital, Capital Medical University, Beijing, China; ^2^ Department of Neurology, The Second Hospital of Lanzhou University, Lanzhou, China; ^3^ Department of Neurology, Beijing Tiantan Hospital, Capital Medical University, Beijing, China; ^4^ DHC Mediway Technology Co. Ltd., Beijing, China

**Keywords:** thrombolysis, acute ischemic stroke, machine learning algorithms, r-tPA, models

## Abstract

**Background:** Thrombolysis with r-tPA is recommended for patients after acute ischemic stroke (AIS) within 4.5 h of symptom onset. However, only a few patients benefit from this therapeutic regimen. Thus, we aimed to develop an interpretable machine learning (ML)–based model to predict the thrombolysis effect of r-tPA at the super-early stage.

**Methods:** A total of 353 patients with AIS were divided into training and test data sets. We then used six ML algorithms and a recursive feature elimination (RFE) method to explore the relationship among the clinical variables along with the NIH stroke scale score 1 h after thrombolysis treatment. Shapley additive explanations and local interpretable model–agnostic explanation algorithms were applied to interpret the ML models and determine the importance of the selected features.

**Results:** Altogether, 353 patients with an average age of 63.0 (56.0–71.0) years were enrolled in the study. Of these patients, 156 showed a favorable thrombolysis effect and 197 showed an unfavorable effect. A total of 14 variables were enrolled in the modeling, and 6 ML algorithms were used to predict the thrombolysis effect. After RFE screening, seven variables under the gradient boosting decision tree (GBDT) model (area under the curve = 0.81, specificity = 0.61, sensitivity = 0.9, and F1 score = 0.79) demonstrated the best performance. Of the seven variables, activated partial thromboplastin clotting time (time), B-type natriuretic peptide, and fibrin degradation products were the three most important clinical characteristics that might influence r-tPA efficiency.

**Conclusion:** This study demonstrated that the GBDT model with the seven variables could better predict the early thrombolysis effect of r-tPA.

## Introduction

Acute ischemic stroke (AIS) is a disturbance in cerebral blood flow and has been the leading cause of serious disability and death worldwide (Khandelwal et al., 2016; Kamel et al., 2020). As an acute emergency, AIS is estimated to lead to a loss of 1.9 million neurons per minute, causing irreversible brain injury around the infarction area (Saver, 2006). Timely treatment to remove the obstruction and restore the blood flow has been shown to improve the outcomes in AIS patients (Herpich and Rincon, 2020).

Intravenous thrombolysis with r-tPA is widely recommended to be beneficial for patients with ischemic stroke within 4.5 h of symptom onset (Dong et al., 2017; Phipps and Cronin, 2020). Nevertheless, not all patients can benefit from this therapeutic regimen. Only approximately 40% of the treated patients show improvement, whereas other patients still experience a low recanalization rate (Orban-Kalmandi et al., 2021). Thus, predicting the intravenous thrombolytic effect at an early stage is important for both the clinician and patient.

Machine learning (ML) algorithms are a crucial branch of artificial intelligence and can learn from complex data by using computational techniques to find potential characteristics to make predictions (Heo et al., 2019; Rajkomar et al., 2019; Sirsat et al., 2020). Compared to conventional statistics, ML includes multiple unique algorithms, such as the random forest classifier, support vector machine, and extreme gradient boosting (XGBoost), which can allow computers to create a model by iterative learning (Deo, 2015; Wang et al., 2020). As a multidisciplinary approach, ML algorithms are gaining popularity in addressing complex problems of healthcare decision-making, and some studies have demonstrated that ML tools provide better accuracy and discrimination (Kamal et al., 2018; Fan et al., 2021). Kalscheur et al. (2018) reported the use of ML to create a model that can help doctors discriminate clinical outcomes in those accepting cardiac resynchronization therapy and improve the shared decision-making with patients. In addition, Lee et al. (2020) also found that ML algorithms using magnetic resonance imaging features were much more sensitive than human readings in identifying patients after a stroke within the time window for acute thrombolysis.

Although ML algorithms have been widely used in the prediction of stroke outcomes, few studies have reported the predictive value of the thrombolysis effect at 1 h after r-tPA infusion. In this study, we aimed to develop a model using the ML algorithm to predict the potential effect of r-tPA at admission in patients with AIS.

## Methods

### Study Design and Patients

We retrospectively conducted an analysis using a cohort of AIS patients who received intravenous thrombolysis with r-tPA between January 2019 and October 2020 at the Beijing Tiantan Hospital. The inclusion and exclusion criteria were previously reported by Zhu et al. (2021). Briefly, patients were recruited if they met the following criteria: diagnosis with AIS through magnetic resonance imaging or computed tomography and admittance to the emergency department within 4.5 h of symptom onset. Patients with a history of the following conditions were excluded from the study: having received a mechanical thrombectomy and having a large vessel occlusion. The study was approved by the ethics committee of the Beijing Tiantan Hospital. The requirement for written informed consent was waived because of the retrospective nature of the study. Confidential patient information was deleted from the entire data set prior to the analysis.

Laboratory values before r-tPA use were recorded. Other medical records, including demographic data and the NIH stroke scale (NIHSS) score at admission and 1 h after r-tPA infusion, were carefully extracted.

### Machine Learning Algorithms

Missing values were replaced before the development of the ML-based predictive models. In our analysis, the median value and model were employed for continuous variables and dispersive eigenvalues, respectively. Six ML algorithms, including logistic regression (LR), random forest (RF), XGBoost, adaptive boosting (AdaBoost), gradient boosting decision tree (GBDT), and light gradient boosting machine (LGBM) were applied to determine the best performing model. In addition, the recursive feature elimination method was used to calculate the contribution of each of the variables. The most optimal variables were further validated using the GBDT method.

### Model Validation

All patients were randomly divided into training and testing sets at a ratio of 8:2. Once the models were derived, the performances of the different models were validated using the receiver–operating characteristic (ROC) curve as the evaluation metric. The area under the curve (AUC) was calculated to evaluate the performance of the ML algorithm between the training and testing sets. The F1 score was calculated to evaluate the performance of each model. Finally, the optimal ML algorithm was selected.

### Model Interpretation

The Shapley additive explanations (SHAP) value, which was developed from the cooperative game theory, was used to interpret the predictions made by the models. By marginally calculating the contributions of the variables, the SHAP method can better explain the importance of each variable in all factor sequences. The local interpretable model–agnostic explanation (LIME) was also used to explain the predictions. The rationale by which a model predicts a single sample using a local linear approximation of the model behavior can be better trusted.

### Statistical Analysis

According to the NIHSS score at admission and 1 h after r-tPA infusion, all patients were divided into two groups, i.e., those having a favorable effect and those with an unfavorable r-tPA effect. Those with a 1-h NIHSS score of ≤1 point after thrombolysis or a 1-h NIHSS score that decreased by at least four points below the score at admission were considered favorable. By contrast, other effects were considered unfavorable (Guo et al., 2020).

Data are presented as medians with interquartile ranges (IQRs) (for non–normally distributed variables), means with standard deviations, or percentages (%). A Fisher exact test or a χ^2^ test was conducted for binary variables, and a Student t-test or Mann–Whitney *U* test was used for the continuous variables. The six ML algorithms were developed and validated using Python software. Statistical significance was set at *p* ≤ 0.05. Statistical analyses were conducted using Python software (version 3.8).

## Results

AIS patients were divided into favorable and unfavorable effects according to the NIHSS score at admission and 1 h after r-tPA. The baseline characteristics of the patients have been reported elsewhere. Briefly, 353 patients with an average age of 63.0 (56.0–71.0) years were enrolled in the study. Of them 156 patients with an average age of 63.0 (55.0–70.25) years had a favorable effect and 197 patients with an average age of 63.0 (56.0–71.0) years had an unfavorable effect. At admission, the average NIHSS score for the patients with a favorable effect was 4.0 (2.0, 9.0) and for those with an unfavorable effect was 5.0 (3.0, 8.5). About 1 h after receiving rt-PA, the NIHSS scores were 1.0 (0.0, 2.0) and 4.0 (2.0, 8.0) for the favorable and unfavorable effect groups, respectively (Table 1).

**TABLE 1:** Demographic and laboratory data of the AIS patients stratified according to the NIHSS score.

Variable	Total	Favorable prognosis	Unfavorable prognosis	*p*-value
	353	156	197	
Age, y^a^	63.0 (56.0, 71.0)	63.0 (55.0, 70.25)	63.0 (56.0, 71.0)	0.438
BMI, kg/m^2^ ^a^	25.24 (23.05, 27.36)	25.33 (23.03, 27.46)	25.15 (23.12, 27.34)	0.373
Gender				0.095
Male	261 (73.94%)	108 (69.23%)	153 (77.66%)	
Female	92 (26.06%)	48 (30.77%)	44 (22.34%)	
TIA, n (%)				0.045
No	261 (73.94%)	108 (69.23%)	153 (77.66%)	
Yes	70 (19.83%)	39 (25.0%)	31 (15.74%)	
Missing	22			
NIHSS score at admission^a^	5.0 (3.0, 9.0)	4.0 (2.0, 9.0)	5.0 (3.0, 8.5)	0.003
NIHSS score after rt-PA 1 h^a^	3.0 (1.0, 6.0)	1.0 (0.0, 2.0)	4.0 (2.0, 8.0)	<0.001
BNP^a^	35.6 (16.9, 97.28)	28.0 (13.65, 81.75)	42.4 (21.6, 103.4)	0.003
APTT (time)^a^	29.6 (27.2, 31.5)	29.8 (28.18, 31.58)	29.0 (26.7, 31.5)	0.011
RDW-CV, %^a^	12.7 (12.4, 13.2)	12.7 (12.4, 13.1)	12.8 (12.4, 13.2)	0.01
MO, %^a^	5.2 (4.2, 6.5)	5.65 (4.65, 6.73)	5.0 (4.0, 5.9)	0.002
FDP, ng/ml^a^	1.33 (0.96, 2.0)	1.205 (0.88, 1.8)	1.5 (1.03, 2.13)	0.001
MYO, ng/ml^a^	42.0 (30.35–63.65)	38.95 (27.8, 58.58)	44.4 (33.45, 68.0)	0.007
EO, %^a^	1.2 (0.5, 2.2)	1.3 (0.6, 2.4)	1.1 (0.4–2.1)	0.04
GR, %^b^	69.68 ± 12.038	67.04 ± 11.98	71.741 ± 11.98	0.001
Glu, mmol/L^a^	6.84 (5.92, 8.80)	6.44 (5.68, 8.33)	7.27 (6.15, 9.11)	0.003
cTnl, ng/ml^a^	0.003 (0.001, 0.006)	0.002 (0.001, 0.004)	0.003 (0.002, 0.007)	0.001
CK-MB, ng/ml	1.2 (0.8, 1.6)	1.1 (0.8, 1.5)	1.2 (0.8, 1.7)	0.036
D-D, ug/ml^a^	0.6 (0.4, 0.9)	0.6 (0.4, 0.8)	0.61 (0.45, 0.99)	0.012
NLR^a^	2.87 (2.08, 4.63)	2.59 (1.90, 4.16)	3.04 (2.28, 4.79)	0.002

a Values are presented as median (IQR).

b For continuous variables, values are presented as mean ± SD.

c TIA:Transient Ischemic Attacks.

### Model Performance

A total of 14 variables including activated partial thromboplastin clotting time [APTT], monocytes percent (MO), B-type natriuretic peptide (BNP), red cell distribution width (RDW-CV), fibrin degradation products (FDP), glucose (Glu), granulocyte ratio (GR), cardiac troponin I, eosinophil ratio (EO, %), creatine kinase-MB (CK-MB), myoglobin (Myo), D-dimer (D-D), and neutrophil–lymphocyte ratio (NLR) were enrolled in our modeling (Supplementary Material). The AIS patients were randomly stratified (8:2) into the training set for developing the models and the testing set for evaluating the model performance. The clinical characteristics of the two data sets are presented in Table 2.

**TABLE 2:** Patient characteristics divided by training data set and testing data set.

Characteristic	Total (*n* = 353)	Training data set (*n* = 282)	Testing data set (*n* = 71)
Age, y^a^	63.0 (56.0, 71.0)	63.0 (56.0, 70.0)	63.0 (56.0, 72.5)
BMI, kg/m^2^ ^a^	25.24 (23.39, 27.06)	25.24 (23.38, 27.04)	25.249 (23.41, 27.14)
Gender			
Male	261 (73.94%)	208 (73.76%)	53 (74.65%)
Female	92 (26.06%)	74 26.24%)	18 (25.35%)
TIA, n (%)			
No	283 (80.17%)	225 (79.79%)	58 (81.69%)
Yes	70 (19.83%)	57 (20.21%)	13 (18.31%)
BNP^a^	35.6 (17.8, 96.7)	35.6 (17.0, 91.78)	39.3 (19.15, 102.25)
APTT (time)^a^	29.6 (27.2, 31.5)	29.6 (27.33, 31.2)	29.2 (26.95, 31.95)
RDW-CV, %^a^	12.7 (12.4, 13.2)	12.7 (12.4, 13.2)	12.8 (12.4, 13.15)
MO, %^a^	5.2 (4.6, 5.9)	5.2 (4.6, 5.8)	5.2 (4.8, 6.55)
FDP, ng/ml^a^	1.33 (0.97, 2.0)	1.3 (0.94, 1.96)	1.52 (1.025, 2.02)
MYO, ng/ml^a^	42.0 (31.0, 63.0)	42.15 (31.83, 62.53)	38.8 (26.25, 64.0)
EO, %^a^	1.2 (0.5, 2.2)	1.1 (0.43, 2.2)	1.5 (0.6, 2.3)
GR, %^b^	69.36 ± 10.44	69.78 ± 10.27	67.69 ± 10.27
Glu, mmol/L^a^	6.84 (6.23, 7.96)	6.84 (6.19, 8.01)	6.84 (6.23, 7.03)
cTnl, ng/ml^a^	0.003 (0.001, 0.006)	0.003 (0.001, 0.005)	0.003 (0.002, 0.007)
CK-MB, ng/ml^a^	1.2 (0.8, 1.6)	1.2 (0.8, 1.6)	1.1 (0.8, 1.49)
D-D, μg/ml^a^	0.6 (0.41, 0.9)	0.6 (0.43, 0.87)	0.6 (0.4, 0.92)
NLR^a^	2.87 (2.08, 4.63)	2.9 (2.13, 4.67)	2.709 (1.94, 4.34)

a Values are presented as median (IQR).

b For continuous variables, values are presented as mean ± SD.

c TIA:Transient Ischemic Attacks.

**FIGURE 1:**
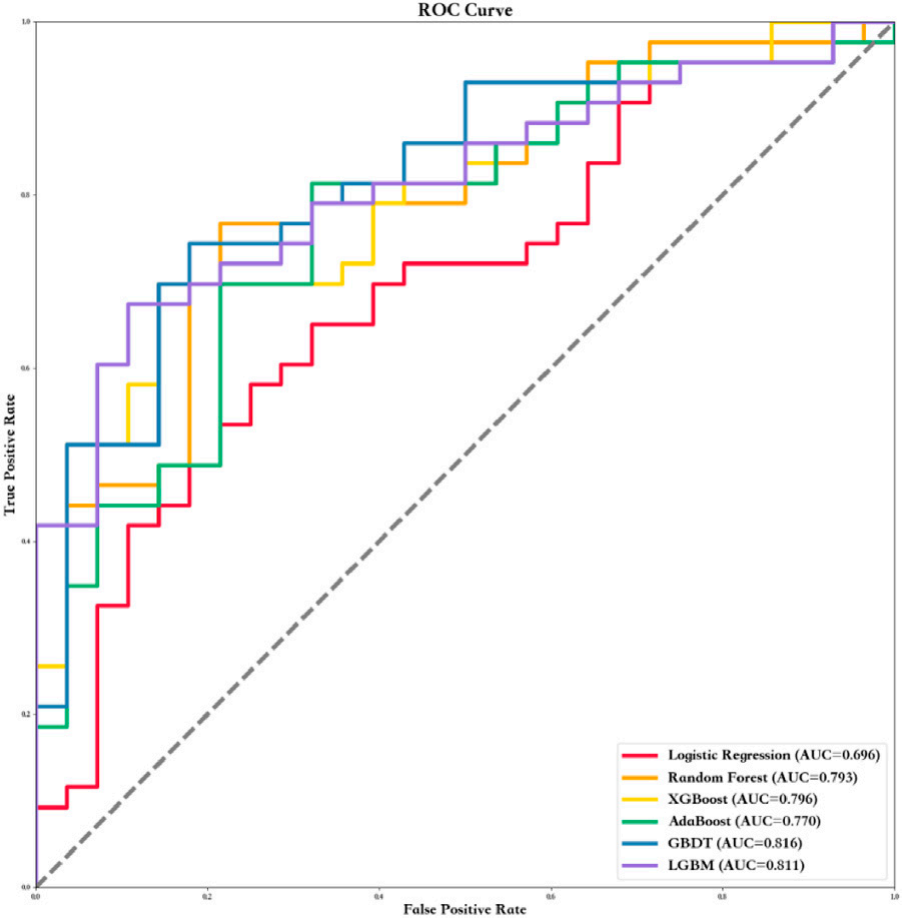
ROC curves of six ML algorithms based on variables in the testing data set.

Six ML algorithms including LR, RF, XGBoost, AdaBoost, GBDT, and LGBM were used to find the model with the best predictive performance. The results showed that the GBDT model demonstrated the best performance (AUC = 0.82, specificity = 0.68, sensitivity = 0.87, and F1 score = 0.80). In addition, the AUC, sensitivity, specificity, F1 score, positive predict (PPV), negative predict (NPV), positive likelihood ratio (PLR), and NLR values of the other five models in the training and testing data sets are shown in Table 3 and Figure 1.

**TABLE 3:** Summary of prediction results of six ML algorithms based on the training data set and testing data set.

Model	LR	RF	XGBoost	AdaBoost	GBDT	LGBM
Train data set AUC (95% CI)	0.58 (0.52, 0.65)	0.8 (0.75, 0.85)	0.88 (0.84, 0.92)	0.842 (0.80, 0.89)	0.97 (0.96, 0.99)	0.96 (0.94, 0.98)
Testing data set AUC (95% CI)	0.70 (0.57, 0.82)	0.79 (0.69, 0.90)	0.80 (0.69, 0.90)	0.77 (0.66, 0.88)	0.82 (0.72, 0.92)	0.81 (0.71, 0.91)
Specificity	0.54	0.69	0.65	0.70	0.68	0.64
Sensitivity	0.78	0.85	0.88	0.80	0.87	0.91
F1	0.75	0.77	0.77	0.78	0.80	0.79
Youden index	0.32	0.53	0.53	0.50	0.54	0.55
NPV	0.75	0.79	0.86	0.68	0.82	0.89
PPV	0.58	0.77	0.70	0.81	0.74	0.67
PLR	1.69	2.71	2.51	2.69	2.67	2.53
NLR	0.41	0.22	0.18	0.29	0.20	0.15

**FIGURE 2:**
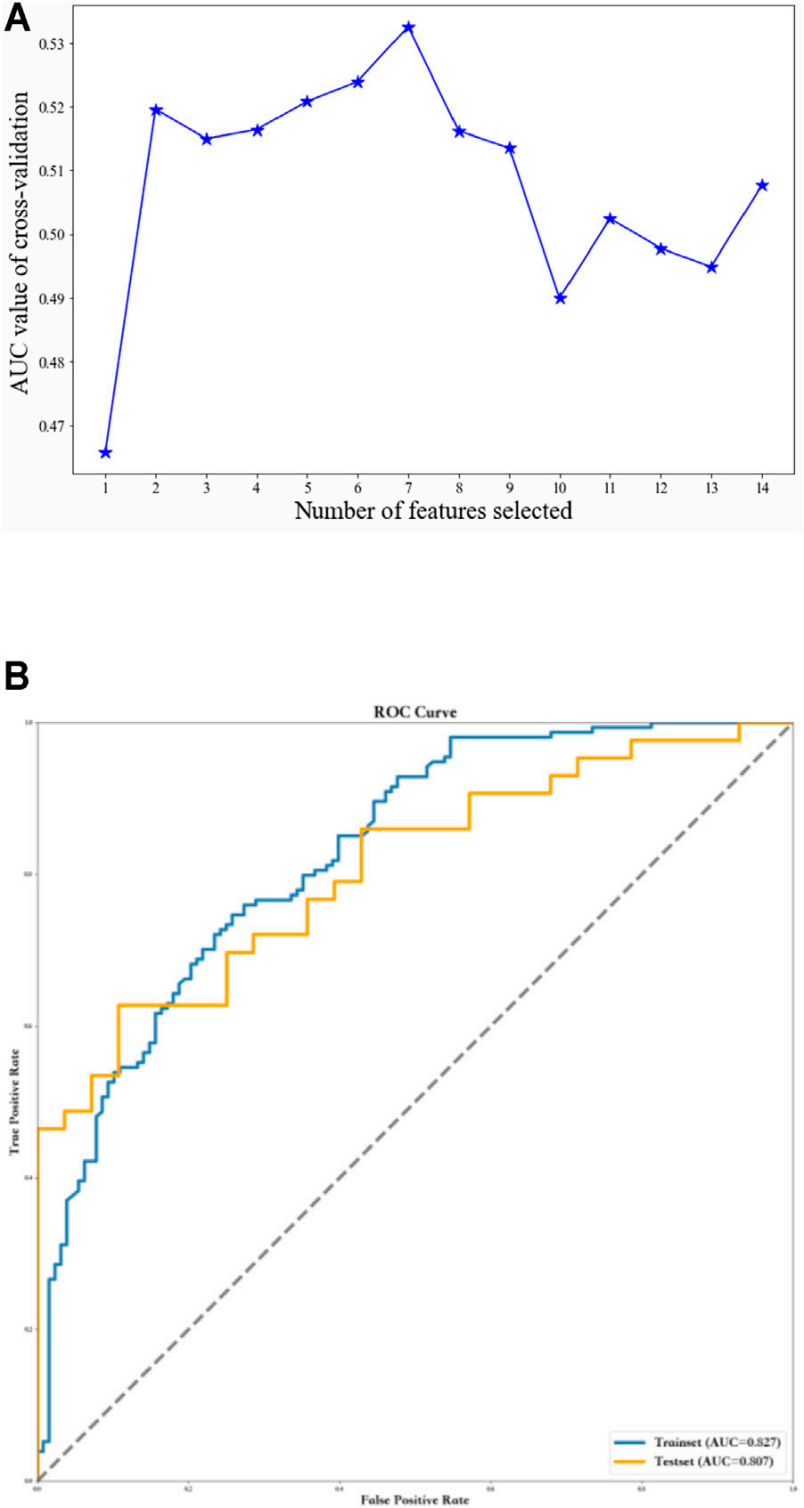
Using recursive feature elimination method to screen the optimal variables. **(A)** Seven variables were employed for the optimal GBDT algorithm. **(B)** ROC curves of the GBDT model based on selected variables.

To further optimize the GBDT model, all 14 samples were filtered individually using the F method. Finally, seven variables were employed for the optimal GBDT algorithm (AUC = 0.81, specificity = 0.61, sensitivity = 0.9, and F1 score = 0.79) (Table 4; Figure 2A,B).

**TABLE 4:** Results of LIME with GBDT model under seven most important variables. Four patients were random selected to interpret of sample prediction results using true negative, true positive, false negative, and false positive.

Model	GBDT
Train data set AUC (95% CI)	0.83 (0.78, 0.87)
Testing data set AUC (95% CI)	0.81 (0.71, 0.91)
Specificity	0.61
Sensitivity	0.9
F1	0.79
Youden index	0.51
NPV	0.89
PPV	0.63
PLR	2.31
NLR	0.16

### Interpretation and Evaluation of Machine Learning Model

**FIGURE 3:**
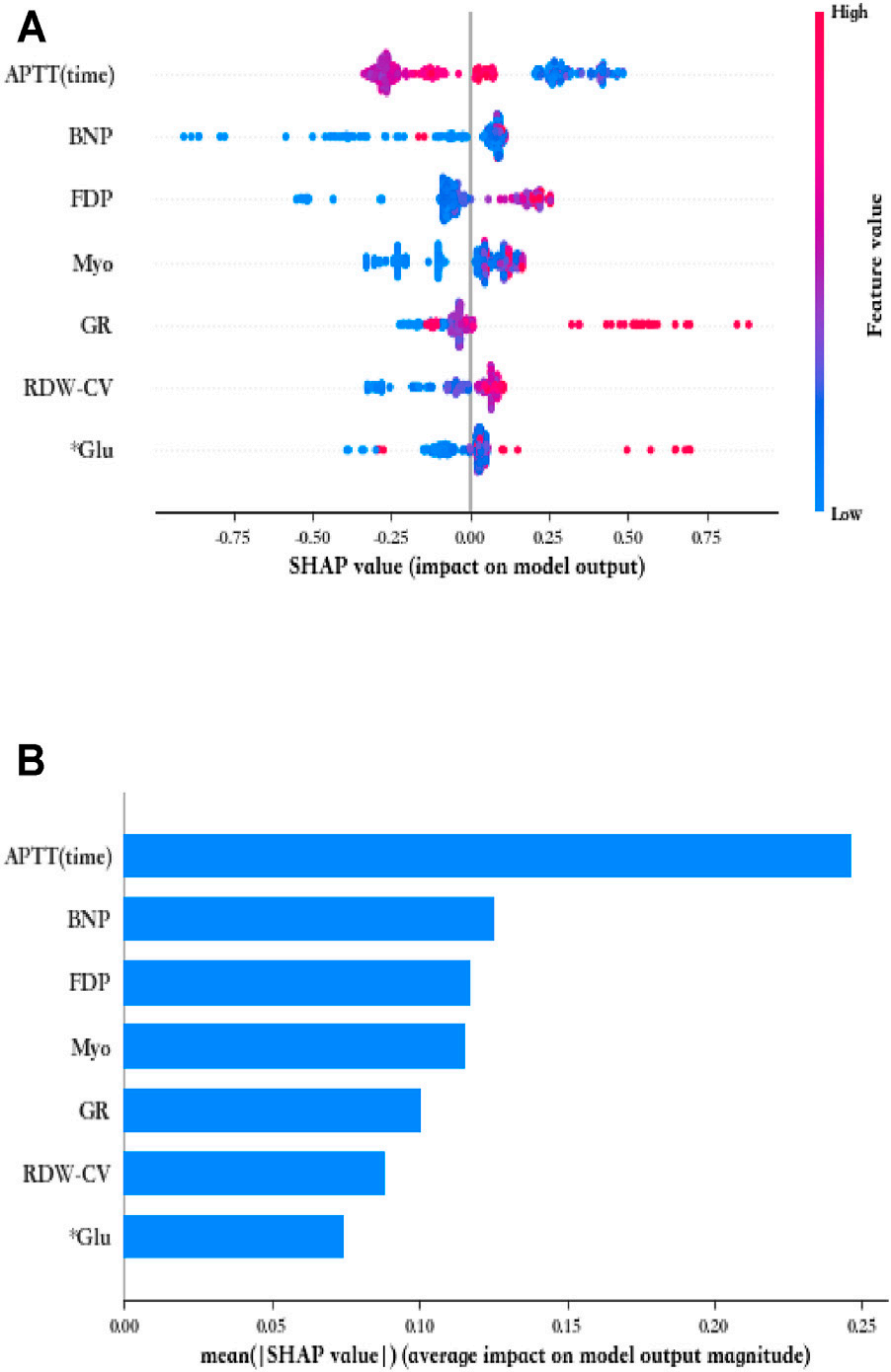
Seven most important variables and their impact on the GBDT model output by SHAP analysis. **(A)** Summary of SHAP analysis on the data set. One dot represents a case in the data set, and the color of a dot indicates the value of the feature. Blue indicates the lowest range and red the highest range. **(B)** Ranking of the seven variables' importance indicated by SHAP analysis.

The SHAP method was also used to interpret the predictions achieved by the GBDT model. Based on the SHAP results, the feature ranking interpretation showed that APTT, BNP, and FDP were the three most important features. Overall, the characteristics of Myo, RDW-CV, FDP, BNP, Glu, and GR correlated positively with the outcomes, indicating increased risk; in addition, the effect of APTT on the patient outcome is nonlinear and fluctuates (Figure 3).

**FIGURE 4:**
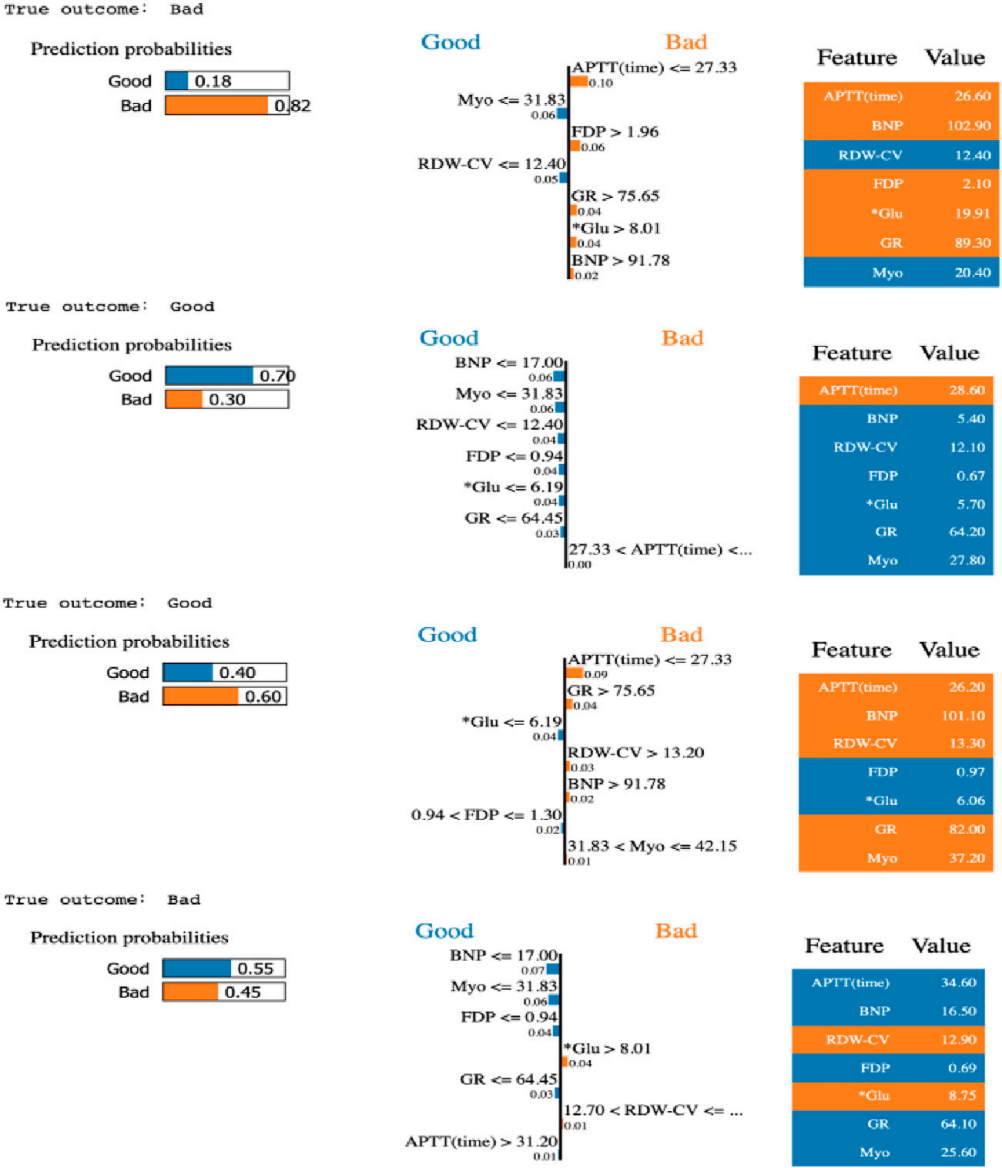
Results of LIME with GBDT applied to four random selected patients.

Moreover, the LIME algorithm was applied to explain the influence of different variables of the GBDT model on the prediction results. Four cases (true positive, true negative, false positive, and false negative) in the testing data set were randomly selected to interpret the visualized prediction results (Figure 4).

## Discussion

Extensive efforts have been made to stratify the long-term outcomes of AIS after r-tPA (Di Lorenzo et al., 2021; Goh et al., 2021). However, early intervention for AIS is particularly important as a disease with a high mortality and disability rate. Thus, this study was designed to evaluate the risk factors which may influence the thrombolytic effect of r-tPA in the early stage of AIS by using ML algorithm. Our study using GBDT model demonstrated that Myo, RDW-CV, FDP, BNP, Glu, and GR are risk factors and should be considered before thrombolysis is applied in clinical scenarios. As an important tool in artificial intelligence, ML has recently received increasing attention and is widely utilized in medical data processing. One of the advantages of ML is the development of predictive models for prognostic outcomes among various confounding factors. Ou et al. (2020) reported a positive result in aneurysm rupture risk assessment using the ML method when compared with a conventional statistical model. Sung et al. (2020) found that ML algorithms that integrate clinical and neuroimaging information can better predict early neurological deterioration in patients after an acute minor stroke. Although these studies showed a good performance through the use of ML, they were not aimed at predicting the super-earlier therapeutic response of r-tPA. To the best of our knowledge, this is the first study to use ML algorithm to predict the 1-h clinical outcome of AIS patients after r-tPA thrombolysis.

Making an accurate prediction under a clinical scenario is usually difficult owing to the existence of too many potential heterogeneous factors and the inherent noise of data encountered in clinical care. Thus, it is preferable to use a simple model if it is sufficiently accurate for a particular application. In our study, we used both 14 and seven variables during the modeling. Both showed good prediction efficiency; however, because the use of seven variables is more feasible under real clinical scenarios, we applied the seven variables to the GBDT model.

Although ML algorithms enable a computer to process complex calculations between variables and outcomes to achieve a more relevant prediction, some aspects of ML algorithms, such as their “black box” characteristic, have limited their predictive value (Petch et al., 2021). Owing to the black box characteristic, ML algorithms are considered to lack a transparent interpretation of the learning. Thus, an ML algorithm model should be used along with the experiences of the physician, rather than as a clinical judgment tool.

To overcome the weaknesses of ML, the SHAP method, which employs a game theory–based approach, was used to interpret the predictions made by the best model. As a unified framework that improves the interpretability and maintains the predictability of complex models, the Shapley value can provide insight for determining the relationships among numerous variables. By using the SHAP method, we identified the characteristics of the important variables that contribute to a prediction, including APTT (time), Myo, and BNP.

The present study has several limitations that should not be ignored. First, this was a single-center retrospective study with a small sample size, which may influence the model efficiency. Further validation in a multicenter setting is required. Second, only the NIHSS score at 1 h was employed in the analysis to evaluate the thrombolytic effect, and long-term follow-up results should be added in the future study. Third, further validation and assessment of the model is required. Because AIS is a complex disease which may be influenced by various factors (such as disease history and home therapies), the analysis of this study was mainly based on the NIHSS score, which may not be enough to accurately predict the thrombolysis effect.

## Conclusion

In summary, this study demonstrated the feasibility of applying ML algorithm in predicting the curative effect 1 h after thrombolysis. However, further studies with a larger cohort are needed to validate the model accuracy in the future, and the predictive value of the model needs to be further examined in clinical practice.

## Data Availability

The original contributions presented in the study are included in the article/Supplementary Material; further inquiries can be directed to the corresponding authors.
