# Supplementary material for: Predicting 1-Hour Thrombolysis Effect of r-tPA in Patients With Acute Ischemic Stroke Using Machine Learning Algorithm
Source: Front Pharmacol. 2022 Jan 3;12:759782. doi: 10.3389/fphar.2021.759782 (PMC8762247; doi:10.3389/fphar.2021.759782)
Supplement: Supplementary file 1 [file DataSheet1.DOCX]

**Figure 1** Correlation coefficient matrix heat maps showing the Spearman correlations between varibles and outcomes. The darker the color, the stronger the correlation.

**
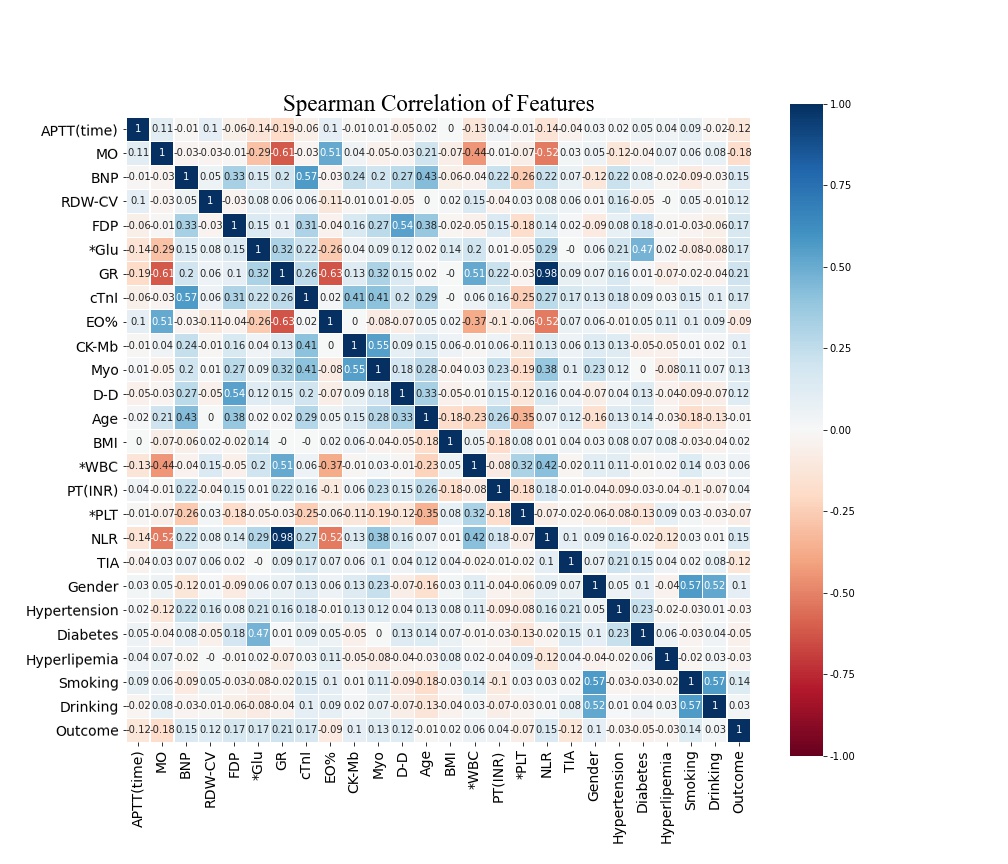
**
